# Stable Isotope-Resolved Metabolomics Shows Metabolic Resistance to Anti-Cancer Selenite in 3D Spheroids versus 2D Cell Cultures

**DOI:** 10.3390/metabo8030040

**Published:** 2018-07-10

**Authors:** Teresa W.-M. Fan, Salim S. El-Amouri, Jessica K. A. Macedo, Qing Jun Wang, Huan Song, Teresa Cassel, Andrew N. Lane

**Affiliations:** 1 Center for Environmental and Systems Biochemistry, Markey Cancer Center, and Depart of Toxicology and Cancer Biology, University of Kentucky, Lexington, KY 40506, USA; sselam0@uky.edu (S.S.E.-A.); jkar224@uky.edu (J.K.A.M.); huan.song@uky.edu (H.S.); terricassel@gmail.com (T.C.); andrew.lane@uky.edu (A.N.L.); 2 Department of Ophthalmology and Visual Sciences, University of Kentucky, Lexington, KY 40506, USA; qingjun.wang@uky.edu

**Keywords:** selenite, cancer metabolism, A549, PANC1, ^13^C_6_-glucose tracer, 2D cell cultures, 3D spheroids

## Abstract

Conventional two-dimensional (2D) cell cultures are grown on rigid plastic substrates with unrealistic concentration gradients of O_2_, nutrients, and treatment agents. More importantly, 2D cultures lack cell–cell and cell–extracellular matrix (ECM) interactions, which are critical for regulating cell behavior and functions. There are several three-dimensional (3D) cell culture systems such as Matrigel, hydrogels, micropatterned plates, and hanging drop that overcome these drawbacks but they suffer from technical challenges including long spheroid formation times, difficult handling for high throughput assays, and/or matrix contamination for metabolic studies. Magnetic 3D bioprinting (M3DB) can circumvent these issues by utilizing nanoparticles that enable spheroid formation and growth via magnetizing cells. M3DB spheroids have been shown to emulate tissue and tumor microenvironments while exhibiting higher resistance to toxic agents than their 2D counterparts. It is, however, unclear if and how such 3D systems impact cellular metabolic networks, which may determine altered toxic responses in cells. We employed a Stable Isotope-Resolved Metabolomics (SIRM) approach with ^13^C_6_-glucose as tracer to map central metabolic networks both in 2D cells and M3DB spheroids formed from lung (A549) and pancreatic (PANC1) adenocarcinoma cells without or with an anti-cancer agent (sodium selenite). We found that the extent of ^13^C-label incorporation into metabolites of glycolysis, the Krebs cycle, the pentose phosphate pathway, and purine/pyrimidine nucleotide synthesis was largely comparable between 2D and M3DB culture systems for both cell lines. The exceptions were the reduced capacity for de novo synthesis of pyrimidine and sugar nucleotides in M3DB than 2D cultures of A549 and PANC1 cells as well as the presence of gluconeogenic activity in M3DB spheroids of PANC1 cells but not in the 2D counterpart. More strikingly, selenite induced much less perturbation of these pathways in the spheroids relative to the 2D counterparts in both cell lines, which is consistent with the corresponding lesser effects on morphology and growth. Thus, the increased resistance of cancer cell spheroids to selenite may be linked to the reduced capacity of selenite to perturb these metabolic pathways necessary for growth and survival.

## 1. Introduction

It is now clear that mammalian cells grown as two-dimensional (2D) cultures on rigid and treated surfaces may exhibit different behavior and functional properties (such as drug sensitivity) from those in the native microenvironment or in three dimensional (3D) cultures i.e., spheroids and organoids [1,2,3,4]. This could be attributable to the lack of cell–cell and cell–extracellular matrix (ECM) interactions as well as the unrealistic gradients of O_2_, nutrients, growth factors, and treatment agents in 2D cultures [1]. Three-dimensional spheroids are self-assembled compact aggregates of single cell types to enable extensive cell–cell and cell–ECM interactions, whereas 3D organoids add the interactions with other cell types [3,5]. They can circumvent the drawbacks of 2D cell cultures in terms of ECM formation, cell–cell interactions, and physicochemical environment. Spheroids can be generated from mono-cultures spontaneously or in an appropriate matrix such as Matrigel, collagen, hyaluronan, or synthetic polyethylene glycol [5,6,7,8,9,10], while organoids are typically formed from live tissues or multiple cell types in Matrigel or collagen [5,9,11,12,13]. Both spheroids and organoids more closely mimic the natural tissue organization than 2D cultures, while retaining similar experimental flexibility [10,14]. They have been shown to display altered gene expression profiles including those metabolism-related, nutrient oxidation capacity, metastatic capacity induced by proline catabolism, O_2_ consumption/extracellular acidification, and drug response compared with the 2D cell culture counterparts [1,3,4,7,15,16,17,18]. However, our knowledge is still very limited regarding the functional or metabolic mechanisms underlying these differences.

Although multiple scaffolds (e.g., Matrigel, hydrogel) or scaffold-free (hanging drop, micropatterned/U-shape cell-repellent plates) 3D cell culture systems have been developed for culturing cancer cell spheroids [3,5,19], they suffer from technical challenges such as long spheroid formation times with variable efficiency and/or difficult handling for high throughput assays [1]. For metabolomic studies, there are added problems of matrix contamination and/or limitations in scaling-up. Magnetic 3D bioprinting (M3DB), a more recent development in 3D culture system, can overcome these difficulties. This technique utilizes nanoparticles composed of gold, iron oxide, and poly-*L*-lysine to magnetize cells, followed by spheroid assembly under mild magnetic forces [1]. Spheroids are formed reproducibly within minutes to hours in cell repellent plates and are biocompatible for various functional assays including toxicity testing [1,20,21,22,23,24,25,26]. This matrix-free method is particularly amenable for Stable Isotope-Resolved Metabolomic (SIRM) studies [2,27,28,29,30,31,32,33,34,35,36,37] as securing spheroids with magnets enables rapid tracer medium change, sampling at different time points or removal during metabolic quenching. Here, we demonstrate the utility of coupling the M3DB culturing with SIRM in exploring the metabolic mechanism that underlies the increased resistance to anti-cancer sodium selenite in 3D spheroids formed from lung (A549) and pancreatic (PANC1) adenocarcinoma cells versus their 2D cell counterparts.

## 2. Results

### 2.1. Three-Dimensional A549 and PANC1 Spheroids Are More Resistant to Selenite Than Their 2D Cell Counterparts

**Figure 1:**
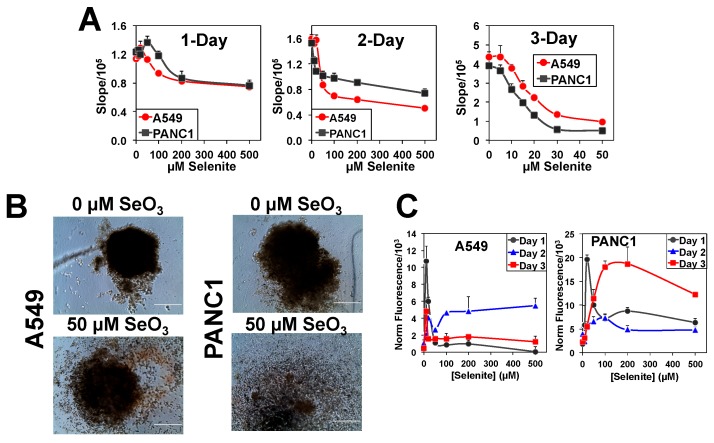
Dose- and time-dependent growth inhibition and ROS production of A549 and PANC1 spheroids by selenite. A549 or PANC1 spheroids were formed, cultured, and assayed for growth using a live cell stain PrestoBlue in 384-well plates as described in Materials and Method. The slope of the time-dependent reduction of PrestoBlue was calculated for each well, averaged, and plotted as a function of selenite concentrations in (**A**); *n* = 5 per treatment. These data were used to estimate IC_50_ and percentage of sensitive cell population in Table 1 by data fitting (see Materials and Methods). In (**B**), example images (10× magnification) of spheroids after 3 days of 0 or 50 µM selenite treatment. Scale bars are 400 μm. In (**C**), time- and selenite dose-dependent production of reactive oxygen species (ROS) by A549 and PANC1 spheroids was measured by dichlorofluoroscein (DCF) fluorescence. *n* = 3 per data point.

We have shown previously that sodium selenite (a chemopreventive agent [38,39]) is toxic to A549 cells in 2D cultures with 24 h IC_50_ (half maximal inhibitory concentration) at 6.25 µM [40]. Here we determined the 26 h IC_50_ of selenite in 2D PANC1 cells to be 10 µM. We also investigated the dose-dependent effect of selenite on the growth of A549 and PANC1 M3DB spheroids. As shown in Figure 1A, selenite had a complex effect on the growth of both spheroid types after 1- and 2-day of treatment. There appeared to be at least two populations of cells with one more resistant than the other. The IC_50_ for neither population could be determined after 1-day treatment. In addition, at doses ≤50 µM, selenite slightly stimulated the overall growth of both spheroid types at 1-day, but not 2-day, of treatment. However, selenite inhibited the growth (Figure 1A) and disrupted spheroid structures (Figure 1B) of both sensitive and resistant populations alike after 3 days of treatment. The complexity of the dose-dependence curves made it difficult to estimate the IC_50_ for the 1-day treatment. We were able to determine the IC_50_ for the sensitive A549 spheroids as 16.7 µM at 3-day and PANC1 spheroids as 9.4 and 13.7 µM at 2- and 3-day, respectively (Table 1). The 2-day IC_50_ for the sensitive A549 spheroids was estimated at 46 µM, which was not as well defined due to insufficient points in the dose-response curve. Suffice to say, the spheroid cultures of A549 and PANC1 cells displayed overall less growth inhibition than the 2D counterparts.

In addition, we measured the ROS production as a function of time and selenite dose in A549 and PANC1 spheroids, as shown in Figure 1C. Selenite induced a burst of ROS production at 10–20 µM after 1 day of treatment in both A549 and PANC1 spheroids. This burst persisted, albeit to a lower extent, for up to 3 days of treatment in A549 but not in PANC1 spheroids. In addition, ROS production did not commensurate with growth inhibition in A549 spheroids but appeared to be related to reduced growth and/or cell death in PANC1 spheroids after 3 days of treatment. Based on these data, we chose 24 h of 10 µM selenite treatment, which gave a burst of ROS in both spheroid types, in subsequent ^13^C_6_-glucose-based SIRM experiments on the spheroid cultures. The results were compared with those obtained from the 2D culture experiments performed with IC_50_ doses of selenite, i.e., 6.25 µM for A549 and 10 µM for PANC1 cells. This design should maximize differential metabolic responses between spheroid (with the resistant cell population presumably unaffected) and 2D cell cultures.

### 2.2. The Higher Selenite Resistance of 3D A549 and PANC1 Spheroids Is Not Due to Less Se Accumulation Than the 2D Counterparts

One immediate question about the higher selenite resistance in the 3D spheroids was if this is due to less Se accumulation in their biomass than that of the corresponding 2D cells. To address this question, we performed parallel selenite treatments of A549 and PANC1 cells as 2D and 3D spheroid cultures for total Se analysis by ICP-MS. We found that total Se in 3D spheroids were higher than that in 2D cultures for both A549 and PANC1 cells (sum, Figure S1). The higher Se accumulation in 3D biomass was a result of elevated selenite uptake from the media for A549 cells but not for PANC1 cells (medium uptake, Figure S3). The latter suggests enhanced loss of absorbed selenite via volatilization [41] in 2D over 3D PANC1 cells. By analyzing Se in polar extracts, protein extracts, and residue (after polar and protein extractions) of A549 and PANC1 cells, we found that the Se content followed the order residue > protein > polar fractions for both 2D and 3D cultures. It is interesting to note that Se was enriched relatively more in the soluble (polar and protein) fractions of 2D than 3D cultures for both A549 and PANC1 cells. It is possible that some form(s) of Se in these fractions contribute to higher toxicity in the 2D cell cultures [40]. In any rate, there was no straightforward relationship between selenite uptake and observed differences in 2D and 3D cell toxicity.

### 2.3. Glycolysis and the Krebs Cycle Respond Less to Selenite in A549 and PANC1 Spheroids than in Their 2D Cell Counterparts

In SIRM experiments, 6.25 µM selenite attenuated net growth and induced morphological changes in A549 cells grown in 2D cultures (Figure S2A). Such changes were less evident in the corresponding 3D spheroids at 10 µM selenite (Figure S2B). Likewise, 10 µM selenite elicited greater perturbations in morphology and proliferation in 2D (Figure S3A) than 3D cell cultures of PANC1 (Figure S3B), which is consistent with the presence of a higher population of resistant cells in spheroid than in 2D cell cultures, as described above. Consistent with the PrestoBlue assay for growth (Figure 1A), the mitotic index (as indicated by the PCNA fluorescence) was reduced by 3 days of 100 µM selenite treatment in both A549 and PANC1 spheroids. The growth inhibition was accompanied by increased necrosis (as indicated by the RIP-1 fluorescence) in both A549 and PANC1 spheroids (Figure S2).

**Figure 2:**
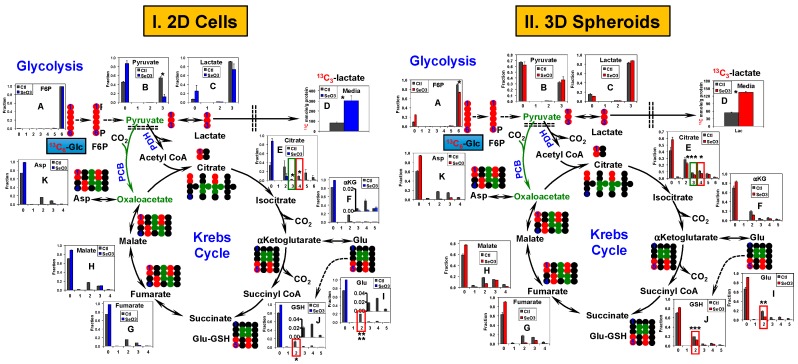
Glycolysis and the Krebs cycle respond less to selenite in A549 spheroids than in their 2D cell counterparts. A549 cells and spheroids were extracted for polar metabolites, which were quantified for the ^13^C isotopologues of various metabolites by IC-UHRFT-MS and for ^13^C_3_-lactate (Lac) by ^1^H-NMR, as described in the Materials and Methods. Oxidation of ^13^C_6_-Glc via glycolysis and Krebs cycle is traced along with the fractional distribution of relevant ^13^C labeled metabolites in control versus selenite-treated 2D cells (**I**) and spheroids (**II**), except for ^13^C_3_-lactate as µmoles/g protein. Not all possible isotopologues are shown. Numbers in X-axis are those of ^13^C atom in each isotopologue. →, ↔⎕, and ---> indicate irreversible, reversible, and multi-step reactions, respectively; double dashed line depicts plasma or mitochondrial membrane; numbers in X-axis refer to those of ^13^C atoms in each isotopologue of metabolites. ●: ^12^C; ●, ●: respective ^13^C fate via the 1st turn of the pyruvate dehydrogenase (PDH)- or pyruvate carboxylase (PCB)-initiated Krebs cycle; ME: malic enzyme; αKG: αketoglutarate; GSH: glutathione; *: *q* (false discovery rate) ≤ 0.05; **: *q* ≤ 0.01; ***: *q* ≤ 0.005; ****: *q* ≤ 5 × 10^−6^. *n* = 2 or 3.

These phenotypic differences were accompanied by differential metabolic responses to selenite between 2D cell and 3D spheroid cultures. Figure 2 compares the extent of ^13^C_6_-glucose (^13^C_6_-Glc) transformation through glycolysis and the Krebs cycle between A549 in 2D culture and as spheroids in response to 24 h of selenite treatments at 6.25 µM and 10 µM, respectively. Significant perturbations to the glycolytic activity were evident in selenite-treated 2D cells based on the enhanced release of ^13^C_3_-lactate into the medium (Figure 2(I-D)) and lower fractional enrichment in intracellular ^13^C_3_-pyruvate (3; Figure 2(I-B)). In contrast, there was no effect of selenite on ^13^C_3_-pyruvate (Figure 2(II-B)) and the stimulation of ^13^C_3_-lactate release was relatively lower from spheroid cultures (Figure 2(II-D)) than from 2D cultures (Figure 2(I-D)); indicating a lesser perturbation of selenite to glycolysis in spheroids than in 2D cells. Likewise, the selenite-induced changes in the Krebs cycle was also much less in spheroids than in 2D cells, as evidenced by the much reduced perturbation to the extent of ^13^C incorporation into the Krebs cycle metabolites, including citrate and *α*-ketoglutarate (*α*KG) (Figure 2(II-E,II-F) versus Figure 2(I-E,I-F), respectively). In particular, the fractional enrichment of ^13^C_4_- (red boxes) and ^13^C_3_-citrate (green boxes) showed prominent differences between the two culture types (Figure 2(I-E,II-E)). As ^13^C_4_-citrate is a marker of pyruvate dehydrogenase (PDH)-initiated Krebs cycle activity [42] while ^13^C_3_-citrate results from pyruvate carboxylase (PCB)-initiated Krebs cycle reactions [34], these differences suggest a differential effect of selenite on both canonical and anaplerotic Krebs cycle activities in A549 spheroids versus 2D cells. Other prominent differences in the selenite effect on the two culture types involved Glu and GSH metabolism. The extent of enrichment in ^13^C_2_-Glu and -GSH (red boxes; Figure 2(I-I,I-J,II-I,II-J)) was much less attenuated in spheroids than in 2D cells. The ^13^C atom-resolved tracing shown in Figure 2 (●, ●) indicates that these two ^13^C isotopologues can be derived from the Krebs cycle reactions initiated by PDH-, PCB-, or both. This agrees with the differential selenite effect on canonical and anaplerotic Krebs cycle activities described above.

**Figure 3:**
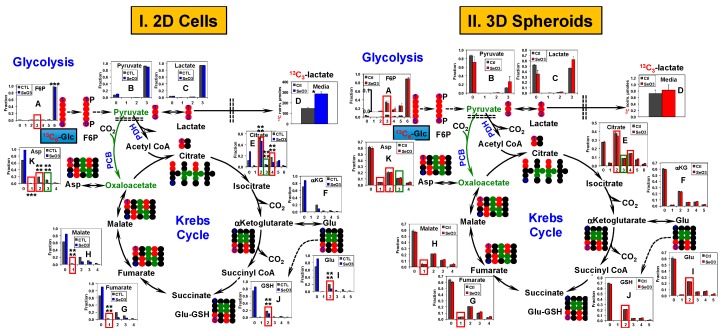
Glycolysis and the Krebs cycle respond less to selenite in PANC1 spheroids than in their 2D cell counterparts. Extraction of polar metabolites and their analysis are as described in Figure 2, so are all symbols and abbreviations. (**I**) Metabolite responses in 2D cultures; (**II**) metabolite responses in 3D cultures.

Similarly, selenite distinctly impacted glycolysis and the Krebs cycle activity in PANC1 2D cell culture (Figure 3I) versus spheroids (Figure 3II). At 10 µM, selenite significantly decreased ^13^C labeling in Krebs cycle metabolites and increased the amount of excreted ^13^C-lactate in the 2D cells but had little effect in the spheroids. The reduced enrichment by selenite in ^13^C_2_-Asp (red box, Figure 3(I-K)) and ^13^C_2_-/^13^C_4_-citrate (red box, Figure 3(I-E); produced in the first and second Krebs cycle turn, respectively [42]) indicated disrupted PDH-initiated Krebs cycle activity while that in ^13^C_3_-Asp and ^13^C_3_-citrate could result from perturbed PCB-initiated Krebs cycle reactions (green box, Figure 3(I-K,I-E)). Again, the reduced enrichment of ^13^C_2_-Glu and -GSH (red box, Figure 3(I-I,I-J)) by selenite is consistent with attenuated PDH- and/or PCB-mediated Krebs cycle activities. However, these selenite-induced perturbations clearly observed in 2D cells (Figure 3(I-E,I,J,K)) were diminished in spheroids (Figure 3(II-E,I,J,K)).

We also noted two clear metabolic differences in PANC1 2D cell and spheroids, regardless of the selenite treatment. One was the higher enrichment in ^13^C_3_-fructose-6-phosphate (F6P) in spheroids (Figure 3(II-A)) than in 2D cells (Figure 3(I-A)). F6P can be produced from ^13^C_3_-pyruvate via gluconeogenesis [35]. The other was the higher enrichment in the ^13^C_1_-isotopologues of fumarate, malate, and Asp in spheroids (Figure 3(II-G,II-H,II-K)) than in 2D cells (Figure 3(I-G,I-H,I-K)). These isotopologues (tracked by ● in Figure S4A) can be produced via the reversible reactions of malic enzyme (ME). Alternatively, these ^13^C_1_-isotopologues can be formed by the condensation of ^13^C_2_-1,2-OAA with unlabeled acetyl CoA and subsequent Krebs cycle reactions, as depicted in Figure S4B (●). If the latter is the case, one would expect the fractional enrichment of ^13^C_1_-fumarate to be higher than that of ^13^C_1_-malate, which was not the case. We hypothesize that ME-mediated reactions contributed at least in part to the production of ^13^C_1_-isotopologues of the Krebs cycle intermediates in PANC1 spheroids.

Thus, spheroid formation led to a higher resistance to selenite toxicity in A549 or PANC1 cells, which was reflected respectively in their attenuated or lack of changes in glycolysis, the Krebs cycle, and GSH metabolism in response to selenite. Additional metabolic rewiring occurred in PANC1 spheroids compared with 2D cultures, most likely involving enhanced gluconeogenesis and malic enzyme activity.

### 2.4. Pyrimidine and the Hexosamine Biosynthetic Pathways Respond Less to Selenite in A549 And PANC1 Spheroids Than in Their 2D Cell Counterparts

**Figure 4:**
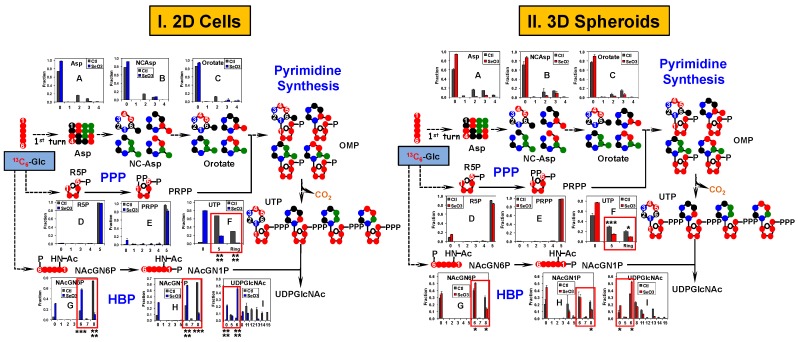
Pyrimidine and the hexosamine biosynthetic pathways respond less to selenite in A549 spheroids than in their 2D cell counterparts. The same polar extracts from Figure 2 were analyzed by IC-UHRFT-MS. ^13^C fate of ^13^C_6_-Glc was followed from the first turn of the Krebs cycle, PPP (pentose phosphate pathway), pyrimidine synthesis, and HBP (hexosamine biosynthetic pathway) into UTP and UDPGlcNAc (UDP-N-acetylglucosamine) along with the fractional distribution of relevant isotopologues of metabolites. ●: ^14^N; NCAsp: *N*-carbamoylaspartate; OMP: orotidine monophosphate; R5P: ribose-5-phosphate; PRPP: phosphoribosylpyrophosphate; NAcGN6P: *N*-acetylglucosamine-6-phosphate; NAcGN1P: *N*-acetylglucosamine-1-phosphate. (**I**) Metabolite responses in 2D cultures; (**II**) metabolite responses in 3D cultures. All other symbols and abbreviations are as in Figure 2.

As Asp is the direct precursor to pyrimidine ring synthesis, which is required for cell proliferation, we then asked if distinct selenite-induced changes of Asp synthesis results in differential inhibition of pyrimidine ring synthesis in 2D versus spheroid cultures of A549 and PANC1 cells. Figure 4 shows the ^13^C enrichment patterns of various precursors and products of uridine synthesis in A549 cells and spheroids including those of the pyrimidine ring and the ribosyl unit, i.e., ribulose/ribose-5-phosphate (R5P) of the pentose phosphate pathway (PPP) and phosphoribosyl pyrophosphate (PRPP). Also tracked were the ^13^C enrichment patterns of the intermediates of the hexosamine biosynthetic pathway (HBP), leading to the synthesis of UDP-N-acetylglucosamine (UDPGlcNAc).

It is clear that the differential ^13^C enrichment patterns of ^13^C_2_-Asp in selenite-treated 2D A549 cells versus spheroids were maintained in the intermediates of the uracil ring synthesis such as *N*-carbamoylaspartate (NCAsp) and orotate (Figure 4(I-B,I-C) versus Figure 4(II-B,II-C), respectively). Subsequent conversion of these ^13^C-labeled precursors into the uracil ring of UTP was also differentially inhibited by selenite in 2D cells (Ring, Figure 4(I-F)) versus spheroids (Ring, Figure 4(II-F)).

In contrast, selenite had a negligible effect on the extent of ^13^C incorporation into R5P and PRPP in both 2D cells (Figure 4(I-D,I-E)) and spheroids (Figure 4(II-D,II-E)). However, differential inhibition in the incorporation of the ^13^C_5_-ribosyl unit of PRPP into UTP by selenite was evident for 2D A549 cells (cf. 5, Figure 4(I-F)) versus spheroids (cf. 5, Figure 4(II-F)). These results suggested that the differential blockade of uracil nucleotide synthesis by selenite is most likely to be mediated via the inhibition of Asp production by the Krebs cycle and of ribosyl incorporation by reactions downstream from PRPP production, e.g., orotate phosphoribosyltransferase.

Furthermore, distinct ^13^C enrichment patterns of the HBP intermediates, *N*-acetylglucosamine-6-phosphate (NAcGN6P, Figure 4(I-G) versus Figure 4(II-G)) and *N*-acetylglucosamine-1-phosphate (NAcGN1P, Figure 4(I-H) versus Figure 4(II-H)) between selenite-treated 2D cells and spheroids of A549 were evident, particularly for the ^13^C_6_- and ^13^C_8_-isotopologues (respectively 6 and 8, Figure 4(I-G,I-H) versus Figure 4(II-G,II-H)). It is interesting to note the opposite effect of selenite on the enrichment patterns of the ^13^C_6_- versus ^13^C_8_-NAcGN6P for both 2D cells and spheroids. Since the synthesis of ^13^C_8_- from ^13^C_6_-NAcGN6P presumably requires ^13^C_2_-acetyl CoA, the large reduction in the extent of ^13^C_8_-NAcGN6P enrichment may reflect reduced synthesis of ^13^C_2_-acetyl CoA via PDH and/or ATP-citrate lyase (ACLY) activity (cf. Figure 2 and Figure S4A; [43]) and/or inhibition in the acetylation of glucosamine-6-phosphate. The former is consistent with the inhibition of the Krebs cycle by selenite described above (cf. Figure 2). The increased fractional enrichment of ^13^C_6_-NAcGN6P was reflected in that of the ^13^C_6_-UDPGlcNAc product for both 2D cells (Figure 4(I-I)) and spheroids (Figure 4(II-I)). This increase could also have contribution from decreased synthesis and/or incorporation of the ^13^C labeled UTP precursor into UDPGlcNAc, as evidenced from the large reduction in the enrichment of ^13^C_11-15_-UDPGlcNAc for both 2D cells and spheroids (11–15, Figure 4(I-I,II-I)). The production of these isotopologues requires ^13^C-labeled UTP (Figure 4) [43]. Further noted was the lower fractional enrichment in these isotopologues in spheroids compared with 2D cells under control conditions, which corresponded to the ^13^C enrichment patterns of the precursors of UTP (Figure 4(I-A–I-F) versus Figure 4(II-A–II-F)) and HBP intermediates (Figure 4(I-G–I-I),versus Figure 4(II-G–II-I)). These results suggest a lower capacity for UTP synthesis (both in terms of the ^13^C_5_-ribosyl incorporation and ^13^C-ring synthesis) and HBP (cf. also higher enrichment of the all ^12^C or 0 isotopologue) in spheroids than in the 2D counterpart.

**Figure 5:**
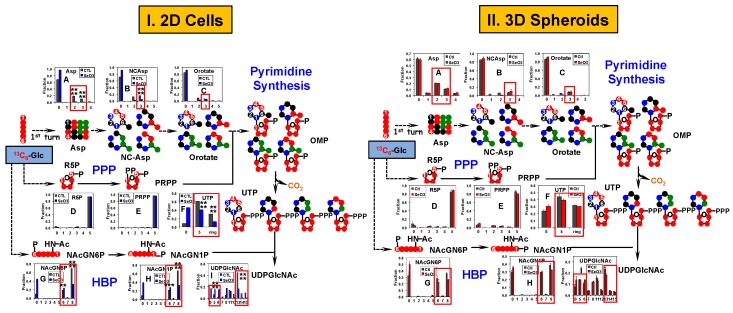
Pyrimidine and the hexosamine biosynthetic pathways respond less to selenite in PANC1 spheroids than in their 2D cell counterparts. The same polar extracts from Figure 3 were analyzed by IC-UHR FT-MS. All symbols and abbreviations are as in Figure 4. (**I**) Metabolite responses in 2D cultures; (**II**) metabolite responses in 3D cultures

Similar to A549 cells, we observed differential selenite inhibition of HBP activity and UTP synthesis, but not of PPP activity and PRPP production in PANC1 2D cell culture (Figure 5I versus spheroids (Figure 5II. Likewise, UDPGlcNAc synthesis was differentially inhibited by selenite in the two PANC1 systems (Figure 5(I-I) versus Figure 5(II-I)). Moreover, intrinsically lower capacity for UTP synthesis and HBP was evident in PANC1 spheroids than 2D cells (cf. higher enrichment of 0 in Figure 5II versus Figure 5I).

## 3. Discussion

Selenite has been investigated extensively for anti-cancer properties in 2D cultures of different cancer cells types including A549 cells [40,44,45,46,47,48,49,50,51]. But virtually nothing is known about its effect on 3D spheroids, which exhibit cell–cell and cell–matrix interactions absent from the 2D cultures. To the best of our knowledge, little is known about the effects of selenite on PANC1 cells either as 2D or spheroid cultures. We showed here that selenite has a complex effect on the spheroid cultures of both A549 and PANC1 cells including responses from a mixed population of resistant and sensitive cells (Figure 1). Although difficult to quantify, it is clear that spheroids were more resistant to selenite than 2D cells when comparing their 1-day IC_50_ values, respectively >100 µM versus 6.25 µM for A549 and >150 µM versus 10 µM for PANC1 (cf. Figure 1, Figures S2 and S3). This resistance of 3D spheroids to selenite toxicity did not result from less Se accumulation into cell biomass (Figure S1). A higher resistance to selenite toxicity with comparable growth rates was also evident for A549 spheroids formed without the magnetic NS (data not shown). These illustrated the influence of the microenvironment, albeit in a simple form, on cancer cells’ response to toxicants. In addition, the selenite toxicity for the sensitive population of PANC1 spheroids with 1-day treatment appeared to be higher than that of A549 spheroids (Figure 1). However, after 2 and 3 days of treatment, the opposite was evident for the selenite toxicity in PANC1 and A549 spheroids (Table 1). Nevertheless, selenite was toxic to both sensitive and resistant populations of A549 and PANC1 spheroids after prolonged exposure.

^13^C_6_-Glc-based SIRM investigations on 1-day selenite treatment of A549 and PANC1 cells and spheroids corroborated with the 1-day IC_50_ trend. Both A549 and PANC1 spheroids displayed less selenite-induced perturbations in the activities of the central metabolic pathways, including glycolysis, the Krebs cycle (Figure 2 and Figure 3), HBP, and pyrimidine ring synthesis (Figure 4 and Figure 5), relative to the 2D cell counterparts. These growth-related metabolic effects are unlikely to be attributed to the presence of NS since NS had a negligible effect on spheroid growth rates and selenite-induced growth attenuation, as indicated above. In addition, the relatively lesser inhibition of these pathways in PANC1 than A549 spheroids was consistent with the lower growth attenuation of the former. Together, these results are consistent with the requirement of these pathways for cell growth. It should also be noted that these metabolic changes occurred before appreciable growth inhibition was evident for A549 spheroids (cf. Figure 1 versus Figure 2), making them more sensitive indicators of selenite toxicity. Moreover, it is likely that the metabolic perturbations manifested in both 3D spheroids resulted from the response of the sensitive populations.

The disruption of GSH synthesis by selenite in 2D A549 cells (Figure 2(I-J)) presumably compromises anti-oxidative defense, which can be related to the enhanced production of reactive oxygen species (ROS) and ROS-mediated cell death observed by Park et al. [49] in selenite-treated A549 cells. This could be the case for the sensitive populations in both A549 and PANC1 spheroids, as evident from the burst of ROS production in 1 day of 10–20 µM selenite treatment (Figure 1C). As such, the relatively less attenuated GSH synthesis in A549 and PANC1 spheroids than in 2D cultures could lead to less oxidative damages and better survival for the resistant populations, which could in turn contribute to the higher IC_50_ for selenite in A549 and PANC1 spheroids than those in the 2D cell counterparts. However, the much lower ROS production in A549 than PANC1 spheroids elicited by the 3-day treatment of selenite suggests that excess ROS was not the key to cell death in A549 spheroids (cf. Figure S2C). Two alternative possibilities warrant further investigation in terms of selenite’s toxic mechanism in A549 spheroids, e.g., (1) inhibition of glutaminolysis via enhanced GLS1 degradation [52]; (2) altered detoxification mechanism involving direct interaction of selenite with GSH to form selenodiglutathione (GSSeG) [53] and subsequent efflux of GSSeG from cancer cells [54].

Besides differential metabolic responses to selenite for spheroids versus 2D cells, we also noted their intrinsically distinct metabolic activities, i.e., reduced capacity for lactate release, UTP synthesis and HBP in both A549 and PANC1 spheroids (Figure 4 and Figure 5), and activation of gluconeogenesis in PANC1 spheroids (Figure 3). Attenuated lactate release by A549 spheroids (Figure 2(II-D)) versus Figure 2(I-D)) could be related to the differences in growth rates (2.9 days for spheroids versus 1.2 days for 2D cells in doubling time). But, this is unlikely to be the case for PANC1 spheroids (Figure 3(II-D)) versus Figure 3(I-D)) since their doubling time (0.6 days) was shorter than 2D cells (1.8 days). Reduced UTP synthesis presumably led to a lower capacity for UDPGlcNAc synthesis in spheroids than 2D cells. UDPGlcNAc is required for O-linked *N*-acetylglucosamine modification (*O*-GlcNAcylation) of regulator proteins, including those important for cancer development and survival [55,56,57,58,59]. It is possible that this difference in capacity for *O*-GlcNAcylation of proteins between spheroids and 2D cells is involved in modulating their differential responses to selenite toxicity. Further investigations will be required to test this hypothesis. It is also foreseeable that enhanced capacity for gluconeogenesis in PANC1 spheroids could improve their survival in a hostile hypoxic tumor microenvironment, where glucose is depleted due to its increased glycolytic conversion to lactate and extracellular release of lactate [60]; the latter response was observed for selenite treatment in the more sensitive A549 spheroids (Figure 2(II-D) versus Figure 3(II-D)). Again, we plan to investigate this hypothesis in future studies.

In summary, both NSCLC and PDAC cancer cell spheroids displayed much higher resistance to anti-cancer selenite toxicity than their 2D cell counterparts in terms of cell growth, which was associated with attenuated response of growth- and survival-requiring central metabolic activities. This resistance can be attributed, at least in part, to the presence of a resistant cell population, presumably as a result of cell–cell and cell–matrix interactions in the spheroids. These interactions also led to reduced intrinsic capacity of UDPGlcNAc synthesis in A549 and PANC1 spheroids and activation of gluconeogenesis in PANC1 spheroids, which could be involved in modulating cancer spheroid metabolism and survival.

## 4. Materials and Methods

### 4.1. IC_50_ Determination for Selenite Treatment of 2D PANC1 Cells

IC_50_ for sodium selenite (Na_2_SeO_3_; Sigma S-1382) in PANC1 cells were initially determined in 96-well plates and further determined in 6-well plates. For 6-well plate (Greiner Bio-one CELLSTAR^®^ 657160), PANC1 cells were grown in DMEM (Sigma D5030; supplemented with sodium bicarbonate 3.7 g/L (pH 7.2), 2 g/L glucose, 2 mM l-glutamine, 10% FBS, 100 U/mL penicillin and 100 μg/mL streptomycin) for two days (reaching 60~70% confluency) before titrating with 0, 2, 10, 20, 50, 200 μM sodium selenite for 26 h. At the end of the treatment, cell viability was determined by neutral red assay. In brief, cell media were aspirated and cells were incubated in 2 mL neutral red solution (33 μg/mL in DMEM) at 37 °C and 5% CO_2_ for 3 h. At the end of the incubation, neutral red solution was aspirated and cells were washed with 2 mL PBS twice. After removing PBS, cells were lysed in 1 mL lysis buffer containing 50% ethanol and 1% acetic acid in water. Lysates were transferred to a 96-well plate (125 μL/well, in triplicates) and A_540_ was measured in a plate reader. Dose response curves were plotted using A_540_ with background subtraction and normalization to control cells. IC_50_ was deduced from curve fitting using SigmaPlot.

### 4.2. Spheroid Formation, Culturing, and Growth Assay

A549 or PANC1 cells were grown in 6-well plates to 50–70% confluence in DMEM growth medium at 37 °C/5% CO_2_ before treating cells with NanoShuttles^TM^-PL (NS, n3D, Biosciences, Inc., Houston, TX, USA) at 1 µL/10^4^ cells overnight according to the vendor’s protocol. NS-loaded cells were then trypsinized, counted, and seeded in a cell-repellent round-bottom 384-well plate at 1000 cells per well. We have separately determined this cell seeding density to be within the linear range of spheroid growth as a function of cell seeding density. The plate was held below a 384-well spheroid drive (n3D) initially for 15 min in the biosafety hood and followed by 45 min at 37 °C/5% CO_2_ for spheroid formation. The drive was then removed and spheroids were allowed to grow for 3 days before medium change to DMEM containing 0 to 500 µM selenite. Spheroid growth was assayed daily using the PrestoBlue^®^ live cell fluorescent stain reagent (Thermofisher, Waltham, MA, USA) [61], which enables in situ monitoring of cellular reducing activity. PrestoBlue^®^ reduction was followed for 30 min at 3–5 min intervals for each well in the 384-well plate using a Cytation 3 imaging microplate reader (BioTek, Winooski, VT, USA) set at 37 °C with 5% CO_2_. The slope of the fluorescence versus time was used to plot the selenite dose-response curve (cf. Figure 1A), from which IC_50_ for the selenite treatment was estimated. IC_50_ values were determined by non-linear regression analysis using Kaleidagraph (Synergy Software, version 4.5) as the Hill equation or the Hill equation modified to two components (sensitive and resistant).

y = y_min_ + D/[1 + (c/IC_50_)^n^]

where D is the change in the number of cells, y_min_ is the plateau value at high concentrations, c, of the inhibitor and n is the Hill coefficient. For biphasic curves, this equation was modified as follows:

y = y_min_ + D/[1 + (c/IC_50_)^n^] − a.c

where a represents the slope of the “resistant” cell population.

### 4.3. ^13^C SIRM Experiments for 2D Cells and Spheroids

2D cell experiments were performed in 10-cm plates with cells grown to 50–70% confluence in DMEM growth medium.

PANC1 (passage 11, 5 × 10^5^) cells were seeded on 10-cm plates and cultured in DMEM growth medium using dialyzed, exosome-depleted FBS. Exosome-depleted FBS was prepared by ultracentrifugation at 120,000× *g* for 2 h followed by filtration through a sterile 0.22 μm vacuum filter. Four days after seeding, media were replaced with fresh media containing either 0.2% ^12^C_6_-Glc (unlabeled) or ^13^C_6_-Glc, without or with 10 μM SeO_3_ in triplicates. Aliquots of 200 μL media were collected at 0, 12 and 24 h after treatment started and spun at 3500× *g* for 15 min at 4 °C to remove debris. Aliquots of 100 μL supernatants were subject to protein precipitation by mixing with 400 μL cold acetone, incubating at −80 °C for 30 min before centrifugation at 14,000× *g* for 10 min at 4 °C. Supernatants were aliquoted and lyophilized for NMR analysis. Such acetone extraction method efficiently precipitated proteins and recovered polar metabolites from the media, similarly as the 10% trichloroacetic acid method [40] but without adding salts (Fan, unpublished data). At the end of 24 h treatment, cells were washed in cold PBS, metabolism quenched in cold acetonitrile, and polar/non-polar metabolites extracted in acetonitrile:water:chloroform (2:1.5:1 *v*/*v*/*v*) as described previously [62]. Polar extracts were aliquoted and lyophilized for NMR and IC-UHR FT-MS analyses. The protein pellets were washed in methanol, dried in Vacufuge (Eppendorf, Hamburg, Germany), and re-dissolved in SDS buffer containing 62.5 mM Tris (pH 6.8), 2% SDS and 1 mM DTT using mini-pestles. Protein concentrations were measured by microBCA assay (Pierce Chemical, Dallas, TX, USA), per vendor’s protocol. Of note, the metabolic response of PANC1 cells to selenite did not depend on passage numbers or different time of the experiments. This was also the case for A549 cells provided that the passage numbers do not go beyond 30.

To prepare spheroid cultures for SIRM experiments, 464 µL of NanoShuttles^TM^ was added for 16–18 h to the cells grown in a 10-cm plate at 70% confluency. Then, cells were washed twice with IX PBS, detached from the plate using trypsin, and counted using a hemocytometer. The trypsinized cells were resuspended in DMEM growth medium before seeding in 6-well plates at a density of 400,000 cells/well. A 6-well magnetic levitation drive was put on top of the 6-well plate containing cells for 15 min in the biosafety hood. The cells were then transferred to a 37 °C/5% CO_2_ incubator and incubated for a further 45 min before removing the magnetic drive. Cells were then monitored daily and growth media was renewed every two days. After four days of growth, cells were treated with 10 µM selenite and ^13^C_6_-Glc for 24 h. Media at 50 µL aliquots were collected at 0 and 24 h after selenite/^13^C_6_-Glc treatment. At the end of incubation, spheroids were held with the 6-well magnetic holder during medium removal and washed twice with IX ice cold PBS and once with nanopure water, followed by quenching and extraction of polar metabolites twice each with 1.0 mL 70% methanol/well. This simultaneous quenching/extraction method is compatible with immunofluorescence analysis and gave reproducible and comparable metabolite profiles as the acetonitrile-based quenching/extraction method described above for 2D cell cultures (Fan et al., unpublished data). The method efficiently quenches the hydrolysis of high-energy metabolites as evidenced by the high energy charge ratios calculated for A549 (ca. 0.9) and PANC1 (0.98) spheroids. The polar extracts were lyophilized overnight and subjected to IC-UHR FT-MS analysis. The cell residues were extracted and analyzed for proteins as described above. Polar metabolites from the media were extracted in 80% acetone as described above.

### 4.4. IC-UHR FT-MS Analysis

Ion chromatography-ultra high-resolution Fourier transform-MS (IC-UHR FT-MS) was performed as previously described [33]. Briefly, polar extracts were reconstituted in 20 μL nanopure water, and analyzed by a Dionex ICS-5000+ ion chromatograph interfaced to an Orbitrap Fusion Tribrid mass spectrometer (Thermo Fisher Scientific, San Jose, CA, USA) operating at a resolution setting of 500,000 (FWHM at *m*/*z* 200) on MS1 acquisition to capture all ^13^C isotopologues. The chromatograph was outfitted with a Dionex IonPac AG11-HC-4 µm RFIC&HPIC (2 × 50 mm) guard column upstream of a Dionex IonPac AS11-HC-4 µm RFIC&HPIC (2 × 250 mm) column. Chromatography and mass spectrometric settings were the same as described previously [30] with an acquisition *m*/*z* range of 80 to 700. Metabolites and their isotopologues were identified by chromatographic retention times and their *m*/*z* values compared with those of the standards. Peak were integrated and the areas exported to Excel via the TraceFinder 3.3 (Thermo, Waltham, MA, USA) software package. Peak areas were corrected for natural abundance as previously described [63], after which fractional enrichment and µmoles metabolites/g protein were calculated to quantify ^13^C incorporation into various metabolites.

### 4.5. ^1^H-NMR Analysis of Medium Extracts

Lyophilized medium extracts were redissolved in 35 µL D_2_O containing 8.81 nmoles of *d*_6_-DSS (2,2-Dimethyl-2-silapentane-5-sulfonate, Cambridge Isotope Laboratories, Tewksbury, MA, USA) for 1D ^1^H-NMR analysis using a 1.7 mm inverse triple-resonance HCN cryoprobe on a Bruker AVANCE III NMR at 16.45 T (Bruker Corp., Billerica, MA, USA). A ^1^H 90° pulse with solvent presaturation was used to acquire 1D ^1^H spectra with a 8403 Hz spectral width, 2 s acquisition time, 4 s relaxation delay, and 512 transients. The free induction decays were Fourier-transformed with 1 Hz line-broadening and zero-filled to 131,072 points. The “peak picking” routine in the MestReNova software (Mestrelab Research S.L., Santiago de Compostela, Spain) was used to quantify the ^13^C satellites of the methyl resonance of lactate, which represented ^13^C_3_-lactate based on the splitting patterns. The lactate peak areas were calibrated for nmoles against that of the methyl resonance of *d*_6_-DSS and normalized to the cell protein content. The final ^13^C_3_-lactate content in the media was expressed as mmole/g protein.

### 4.6. Immunofluorescence Measurements

3D Spheroids were suspended in HistoGel (Thermo Scientific HG-4000-012). After solidification, HistoGel pellets were fixed in 10% neutral buffered formalin for 24 h, followed by paraffin embedding, sectioning at 4 μm thickness, and slide mounting. The slides were deparaffinized, rehydrated, subjected to heat-induced epitope retrieval (hier) by microwaving in sodium citrate buffer (10 mM sodium citrate, 0.05% tween 20, pH 6.0) for 10 min at a sub-boiling temperature. For immunofluorescence analysis, slides were treated with AlexaFluor™ Tyramide Superboost™ kits (Invitrogen, Carlsbad, CA, USA) as per manufacturer’s instructions, followed by primary and secondary antibody treatments as described previously [33]. The following primary antibodies were used: PCNA (PC10) mouse mAb (1:4000, Cell Signaling, Danvers, MA, USA, #2586) and RIP (D94C12) XP^®^ rabbit mAb (1:100, Cell Signaling #3493). Slides were mounted in Prolong™ Gold Antifade Mountant with DAPI (Thermo Fisher Scientific P36935) before imaging. Images were acquired at 40× magnification using a laser scanning confocal microscope Olympus Fluoview™ FV1200.

### 4.7. Se Analysis by Inductively-Coupled Plasma-Mass Spectrometry (ICP-MS)

2D and 3D cells were cultured in 6-well plates, as described in 4.2 and 4.3. A549 cells (passage 15) were treated with 6.25 µM (2D) or 10 µM selenite (3D) for 24 h with 200 µL media sampled at 0 and 24 h after selenite treatment. Both 2D and 3D PANC1 (passage 20) cells were treated with 10 µM selenite for 24 h and media sampled as described above. Then 10 µL aliquots of media were digested in triplicate in 0.5 mL concentrated nitric acid (Aristar Plus, VWR chemicals) at 150 °C for 10 min with maximal pressure of 200 psi and power of 150 W using Discover SP microwave digestor (CEM, Matthews, NC, USA). At the end of 24 h treatment, cells were washed in cold PBS, quenched in cold 70% methanol, and extracted for polar metabolites as described above. The protein pellets were then extracted using SDS/Tris buffer and protein concentrations were measured by microBCA assay as above. Both polar and protein extracts were aliquoted in duplicates and lyophilized. The residue pellets were dried in a Vacufuge (Eppendorf) and pre-digested in 100 µL nitric acid overnight. All lyophilized extracts and predigested residue pellets were digested in 250 µL concentrated nitric acid as above and the final digests were diluted to 5% nitric acid with nanopure water. ICP-MS was preformed using an 8800 Triple Quadrupole ICP-MS (Agilent Technologies, Santa Clara, CA, USA) with a micromist nebulizer. Selenium was monitored in MS/MS mode at the second quadrupole as the oxide (SeO, *m*/*z* 96) following collision with oxygen flowing at 50% with integration time of 1 s. Se concentrations in the digests were calculated from a standard curve of sodium selenite (Na_2_SeO_3_) in 5% nitric acid at 5 concentrations from 0.1 to 10 ppm.

## Figures and Tables

**Table 1:** IC_50_ of selenite for A549 and PANC1 spheroids after 2 and 3 days of treatment.

Spheroids	Treatment Days	IC_50_ (µM)	% Sensitive ^a^	*R* ^2^
A549	2	(46) ^b^	53	0.997
A549	3	16.7 ± 0.6	81	0.998
PANC1	2	9.4 ± 0.2	32	0.999
PANC1	3	13.7 ± 0.9	92	0.995

^a^ Percentage of the sensitive cell population (cf. Figure 1A); ^b^ not well-determined.

## Supplementary Materials

The following are available online at http://www.mdpi.com/2218-1989/8/3/40/s1, Figure S1: Higher selenite resistance of A549 or PANC1 spheroids is not due to lower Se accumulation than the 2D counterparts; Figure S2: A549 spheroids respond less to selenite than 2D cultures in terms of morphology, protein content, mitotic index, and necrosis; Figure S3: PANC1 spheroids respond less to selenite than 2D cultures in terms of morphology, protein content, mitotic index, and necrosis.; Figure S4: ^13^C atom-resolved tracing of the synthesis of ^13^C_2_-acetyl CoA and ^13^C_1_-isotopologues of Krebs cycle metabolites from ^13^C_6_-glucose.
