# Supplementary material for: Stable Isotope-Resolved Metabolomics Shows Metabolic Resistance to Anti-Cancer Selenite in 3D Spheroids versus 2D Cell Cultures
Source: Metabolites. 2018 Jul 10;8(3):40. doi: 10.3390/metabo8030040 (PMC6161115; doi:10.3390/metabo8030040)

Supplementary materials

Fig. S1. Higher selenite resistance of A549 or PANC1 spheroids is not due to lower Se accumulation than the 2D counterparts.

A549 cells as 2D and 3D cultures were treated respectively with 6.25 and 10 μM sodium selenite for 24 hr as in **Fig. S1**, while PANC1 cells as 2D and 3D cultures were treated with 10 μM sodium selenite for 24 hr as in **Fig. S2**. The cells were quenched and extracted as in **Fig. 2**. The polar, protein, and remaining residue fractions were obtained and along with the treatment media, they were digested in nitric acid before analysis for total Se content by ICP-MS, as described in the Method. *, p ≤ 0.05; **, p ≤ 0.01; ***, p ≤ 0.005; ****, p ≤ 0.001; n=2.

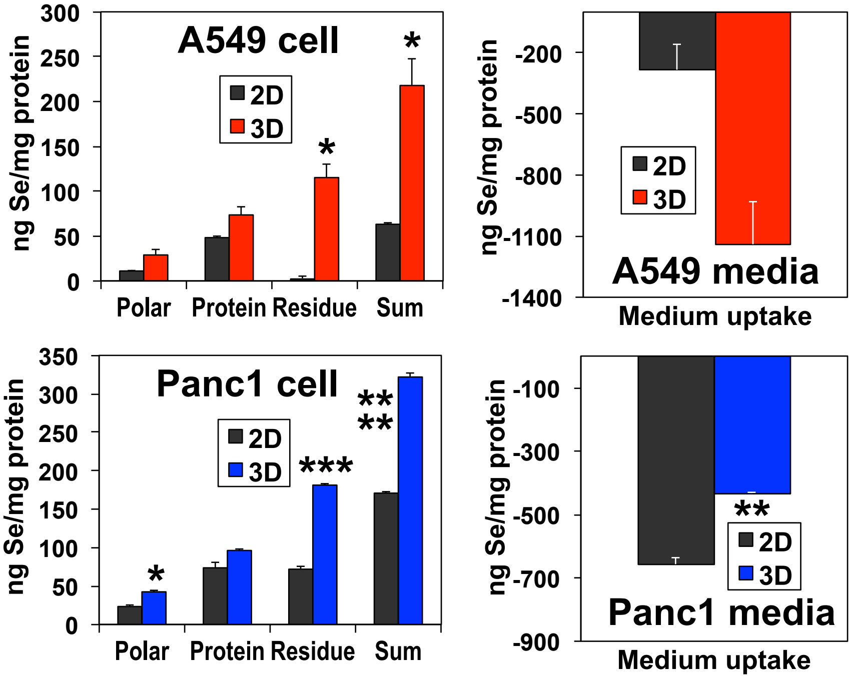

Figure S2. A549 spheroids respond less to selenite than 2D cultures in terms of morphology, protein content, mitotic index, and necrosis.

A549 cells from the 2D (**A**) or spheroid (**B**) SIRM experiments as described in the Materials and Methods were examined for their morphological changes in response to 24 h of 6.25 or 10 μM selenite treatments, respectively. The red arrow in **A** points to vacuole formation in treated 2D cells. Also shown in **A** and **B** are the corresponding changes in extractable protein content from the initial 870,000 and 400,000 cells, respectively. In a parallel experiment, A549 spheroids were subjected to control and 100 μM selenite treatment for 3 days, fixed in 70% methanol, and stained for nuclei using DAPI, mitotic index with PCNA antibody, and necrosis with RIP-1 antibody, as shown in **C**.

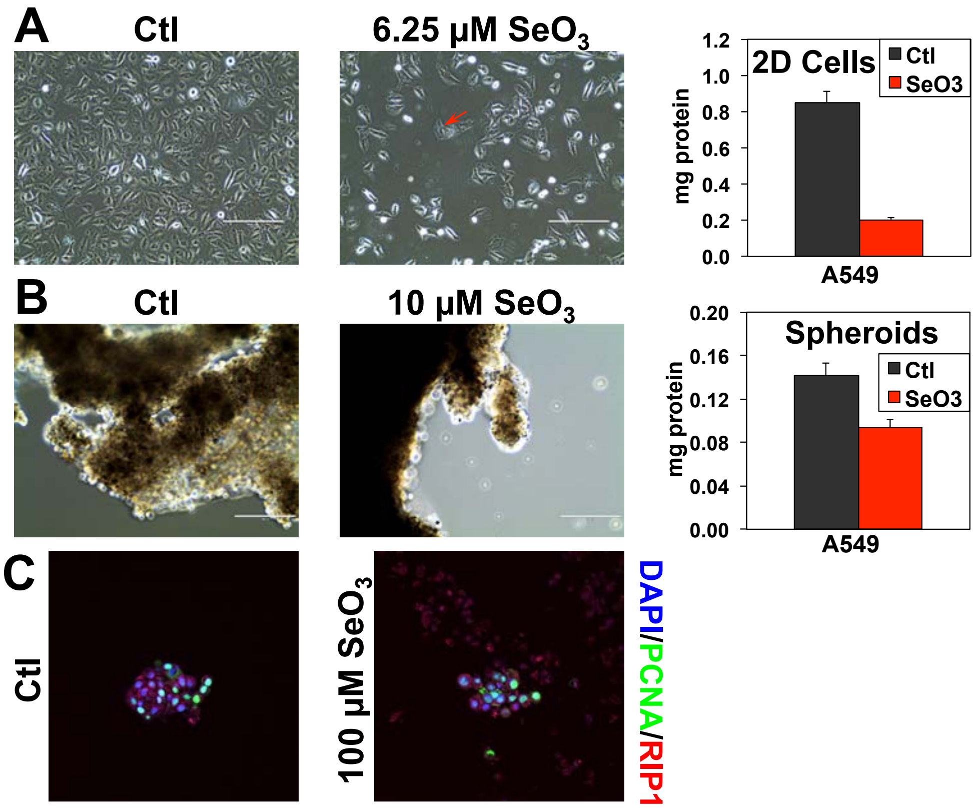

Figure S3. PANC1 spheroids respond less to selenite than 2D cultures in terms of morphology, protein content, mitotic index, and necrosis.

PANC1 cells from the 2D (**A**) or spheroid (**B**) SIRM experiments as described in the Materials and Methods were examined for their morphological changes in response to 24 h of 10 μM selenite treatments. The red arrow in **A** points to vacuole formation in treated 2D cells. Also shown in **A** and **B** are the corresponding changes in extractable protein content from the initial 870,000 and 400,000 cells, respectively. In a parallel experiment, PANC1 spheroids were subjected to control and 100 μM selenite treatment for 3 days, fixed in 70% methanol, and stained for nuclei using DAPI, mitotic index with PCNA antibody, and necrosis with RIP-1 antibody, as shown in **C**.

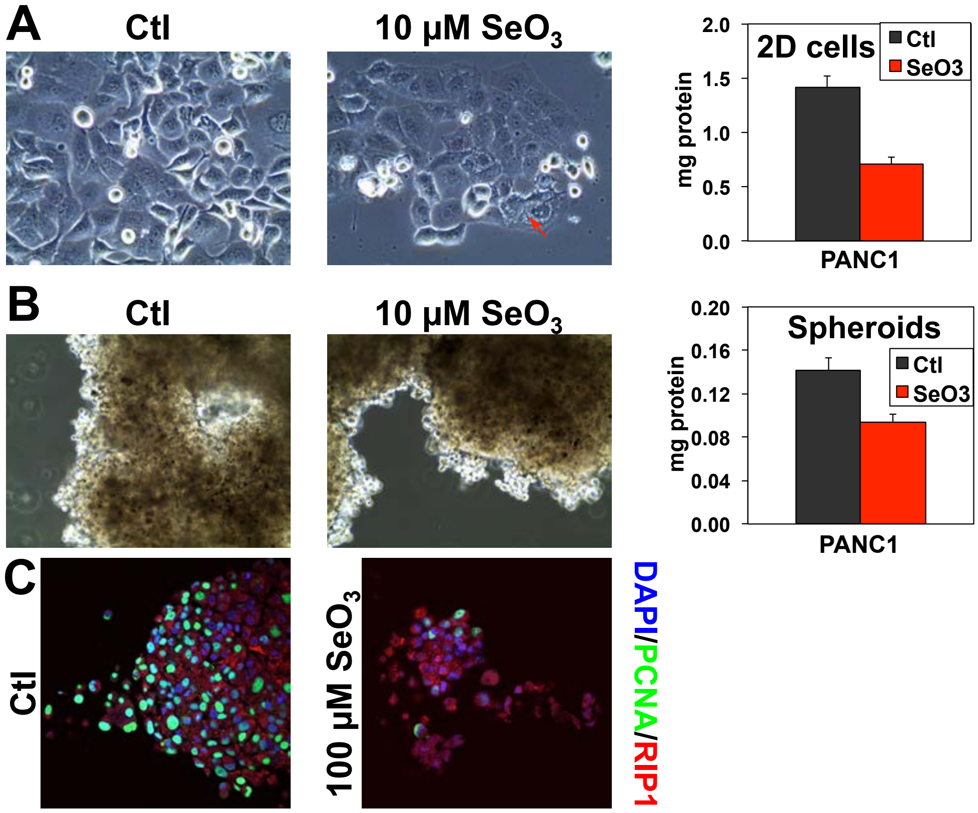

Figure S4. ^{13}C Atom-resolved tracing of the synthesis of $^{13}C_2$-acetyl CoA and $^{13}C_1$-isotopologues of Krebs cycle metabolites from $^{13}C_6$-glucose.

Panel **A** illustrates the synthesis of $^{13}C_1$-malate (denoted by ●) from $^{13}C_6$-Glc via the first turn of the canonical Krebs cycle and reversible reactions of malic enzyme (ME) as well as the production of $^{13}C_2$-acetyl CoA from PDH and ATP-citrate lyase (ACLY) activity. Panel **B** depicts an alternative route of $^{13}C_1$-malate synthesis via condensation of $^{13}C_2$-oxaloacetate derived from the first turn of the Krebs cycle with unlabeled acetyl-CoA, followed by the second turn reactions.

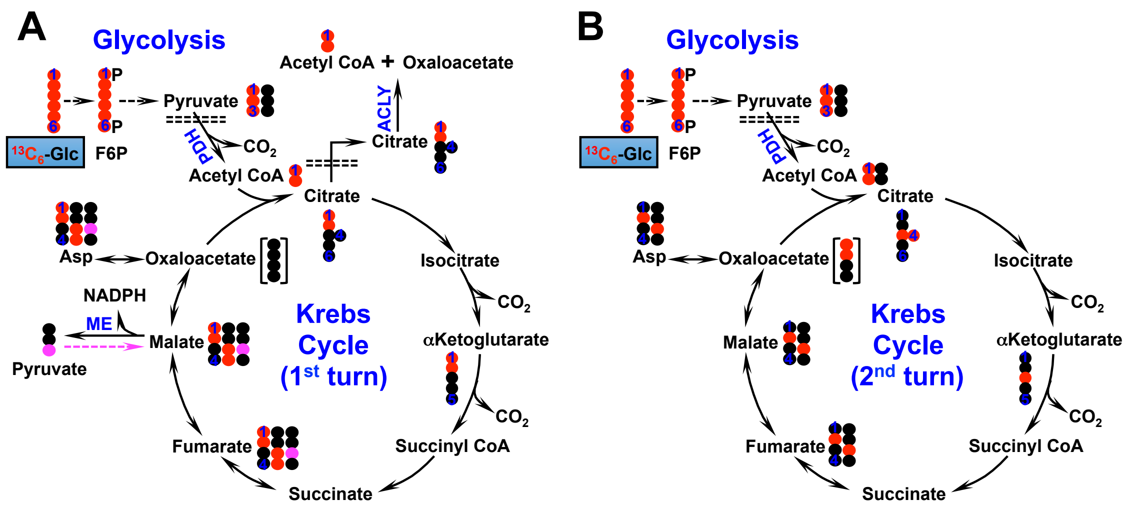

Supplement: Supplementary file 1 [file metabolites-08-00040-s001.pdf]
